# Receptor Activator of NF-κB (RANK) Confers Resistance to Chemotherapy in AML and Associates with Dismal Disease Course

**DOI:** 10.3390/cancers13236122

**Published:** 2021-12-04

**Authors:** Kim L. Clar, Lisa M. Weber, Bastian J. Schmied, Jonas S. Heitmann, Maddalena Marconato, Claudia Tandler, Pascal Schneider, Helmut R. Salih

**Affiliations:** 1 Clinical Collaboration Unit Translational Immunology, German Cancer Consortium (DKTK) and German Cancer Research Center (DKFZ), Department of Internal Medicine, University Hospital Tuebingen, 72076 Tuebingen, Germany; Kim-Larissa.Clar@med.uni-tuebingen.de (K.L.C.); Lisa.Weber@med.uni-tuebingen.de (L.M.W.); bschmied88@googlemail.com (B.J.S.); Jonas.Heitmann@med.uni-tuebingen.de (J.S.H.); Maddalena.Marconato@med.uni-tuebingen.de (M.M.); Claudia.Tandler@med.uni-tuebingen.de (C.T.); 2 DFG Cluster of Excellence 2180 “Image-Guided and Functional Instructed Tumor Therapy (iFIT)”, University of Tuebingen, 72076 Tuebingen, Germany; 3 Department of Biochemistry, University of Lausanne, 1066 Epalinges, Switzerland; Pascal.Schneider@unil.ch

**Keywords:** RANK, AML, chemotherapy resistance, cytarabine, doxorubicin, prognosis

## Abstract

**Simple Summary:**

Acute myeloid leukemia (AML) is the most common form of acute leukemia in adults. Despite the emergence of new therapeutic agents in recent years, curation remains challenging, and new and better treatment options are needed. In the present study, we investigated the expression, prognostic significance, and functional role of the Receptor Activator of Nuclear Factor-κB (RANK) in AML. We found that RANK is expressed on leukemic cells in a substantial proportion of AML patients and is associated with a dismal disease course. We further demonstrated that signaling via RANK induces release of factors that favor AML cell survival and confers resistance to chemotherapeutics in AML treatment. Together, our findings identify RANK as novel prognostic marker and putative candidate for therapeutic intervention in AML to enhance response to treatment.

**Abstract:**

Although treatment options of acute myeloid leukemia (AML) have improved over the recent years, prognosis remains poor. Better understanding of the molecular mechanisms influencing and predicting treatment efficacy may improve disease control and outcome. Here we studied the expression, prognostic relevance and functional role of the tumor necrosis factor receptor (TNFR) family member Receptor Activator of Nuclear Factor (NF)-κB (RANK) in AML. We conducted an experimental ex vivo study using leukemic cells of 54 AML patients. Substantial surface expression of RANK was detected on primary AML cells in 35% of the analyzed patients. We further found that RANK signaling induced the release of cytokines acting as growth and survival factors for the leukemic cells and mediated resistance of AML cells to treatment with doxorubicin and cytarabine, the most commonly used cytostatic compounds in AML treatment. In line, RANK expression correlated with a dismal disease course as revealed by reduced overall survival. Together, our results show that RANK plays a yet unrecognized role in AML pathophysiology and resistance to treatment, and identify RANK as “functional” prognostic marker in AML. Therapeutic modulation of RANK holds promise to improve treatment response in AML patients.

## 1. Introduction

Acute myeloid leukemia (AML) is the most common form of acute leukemia in adults and characterized by a clonal expansion of myeloid precursor cells with a reduced capacity to differentiate [1,2]. Untreated, AML leads to death within months after first symptoms [3]. Combinatorial chemotherapy, mostly using a pyrimidine analog together with an anthracycline, made this previously incurable disease medicable [4], and response to therapy strongly correlates with patient outcome [5]. Nevertheless, curation remains challenging and complications, such as refractory disease and relapse caused by treatment-resistant cells, lead to a poor prognosis with an average 5-year survival rate of 30% [6,7]. Despite the development and approval of several new therapeutic agents in recent years, AML-related deaths are expected to almost double worldwide by 2040 [8]. This underlines the high medical need of patients and the necessity to develop better treatment options based on the discovery of novel druggable targets [9,10].

The members of the tumor necrosis factor (TNF)/tumor necrosis factor receptor (TNFR) superfamily are involved in activation, proliferation, differentiation and cell death of various cell types [11]. The TNFR family member Receptor Activator of Nuclear Factor (NF)-κB (RANK) is best known for its crucial role in regulating bone remodeling [12,13]. In healthy as well as in malignant cells of the hematopoietic system, the RANK/RANKL molecule system was further shown to affect cellular functions [14,15,16], and involvement in metastasis of different cancer entities has been reported [17,18,19,20,21,22]. So far, nothing is known regarding the expression and function of RANK in AML. We here studied the expression of RANK in AML and its functional relevance for leukemia cell cytokine production, survival and treatment resistance. 

## 2. Materials and Methods

### 2.1. Patient Samples

Peripheral blood mononuclear cells (PBMC) collected from blood of AML patients at diagnosis and from bone marrow (BM) of healthy volunteers were isolated by density gradient centrifugation. Informed consent was obtained from all individuals in accordance with the Declaration of Helsinki protocol. Viably frozen cells were freshly thawed prior to each experiment. To avoid potential artifacts by further purification, only PBMCs of patients with ≥85% blast count according to differential blood count in blood smears were used in functional analyses. The study was performed according to the guidelines of the local ethics committee (vote 13/2007V) and all relevant ethical regulations were considered.

### 2.2. Cell Lines

AML cell lines (HL-60, NB-4, THP-1) were obtained from German Collection of Microorganisms and Cell Cultures (Braunschweig, Germany) and tested for mycoplasma contamination by PCR. Authenticity was assessed by routine validation of the respective immunophenotype described by the provider using flow cytometry. 

### 2.3. Quantitative PCR

Amplification of *RANK* cDNA was performed post RNA isolation from AML cell lines and patient samples with ≥85% blast count using the High Pure RNA Isolation Kit (Roche, Mannheim, Germany) and transcription into cDNA using cDNA Synthesis FastGene^®^ Scriptase II 5x ReadyMix (NIPPON Genetics Europe, Dueren, Germany). qPCRBIO SyGreen Mix (PCR Biosystems, London, UK) on a LightCycler^®^ 480 instrument was utilized. The following primers were employed for quantitative PCR of *RANK* (accession number NM_001270949.2; http://www.ncbi.nlm.nih.gov/nuccore/ (accessed on 27 July 2021)), 5′–CCCGTTGCAGCTCAA–3′ and 5′-GCATTTGTCCGTGGAGGAA–3′ (85 bp). 18S ribosomal RNA (*RRN18S*) was detected by Hs_RRN18S_1_SG QuantiTect Primer Assay (Qiagen, Hilden, Germany). Abundance of *RANK* mRNA was calculated using delta-Ct method relative to *RRN18S* expression.

### 2.4. Analysis of the RANK Expression on the Cell Surface

Fluorescent conjugates targeting human (h)RANK (clone 80704, R&D Systems, Minneapolis, MN, USA), CD33, CD117 (both Biolegend; San Diego, CA, USA), CD13 (DAKO, Santa Clara, CA, USA), CD34, 7AAD (both BD Biosciences, Heidelberg, Germany) were used. RANK expression was detected by flow cytometry using mIgG1-PE (BD Biosciences) as isotype control. Analysis was performed on a FACSCanto^TM^ II or a FACS LSRFortessa^TM^ instrument (both BD Biosciences). Specific fluorescence indices (SFI) were calculated as follows: “median fluorescence intensity obtained with specific monoclonal antibody (mAb)” divided by “median fluorescence intensity obtained with isotype control”. Surface positivity was defined as SFI ≥ 1.5.

### 2.5. Cell Viability Assay

PBMC of AML patients were incubated as previously described [23] in the presence or absence of recombinant Fc-hRANKL fusion protein (15 µg/mL, containing amino acids 152-317 of RANKL, produced as described in [24]) or rhIgG1-Fc (R&D) as isotype control for 24 h. Absence of endotoxins in recombinant proteins was confirmed by ENDONEXT^TM^ EndoZyme^®^ II assay (bioMérieux, Nuertingen, Germany) prior to functional analyses. Where indicated, cells were additionally incubated with doxorubicin (1.25 µM) and cytarabine (10 µM; both Selleckchem, Houston, TX, USA) for 24 h and 72 h, respectively. Cell viability was measured as Relative Light Units (RLU) using the CellTiterGlo^®^ Luminescent Cell Viability Assay (CTG) on a GloMax^®^ microplate reader (both Promega, Madison, WI, USA) according to manufacturer’s instructions.

### 2.6. Measurement of Transmembrane Potential and Activation of Caspase-3 

AML patient-PBMC were cultured at 5 × 10^5^ cells per well in a 96-well plate (CELLSTAR^®^ U-Bottom, Greiner Bio-One; Frickenhausen, Germany) and left untreated or were treated with Fc-hRANKL (15 µg/mL) or rhIgG1-Fc as isotype control. After 24 h, therapeutics (doxorubicin, 5 µM; cytarabine, 10 µM) were added as described in the section Cell Viability Assay. Mitochondrial membrane potential was determined in AML cells after incubation with doxorubicin and cytarabine for 24 h and 72 h, respectively, by staining with TMRE (tetramethylrodamine ethyl ester) according to manufacturer’s instructions. For determination of caspase-3 activity in AML cells, PBMC were stained with LIVE/DEAD^TM^ Fixable Aqua (Thermo Fisher Scientific, Waltham, MA, USA). The subsequent intracellular staining of active caspase-3 was performed as previously described [23]. Samples were analyzed on a FACS LSRFortessa^TM^ using a high throughput sampler (both BD Biosciences). Latex beads (Sigma-Aldrich, Darmstadt, Germany) were used to obtain uniform measurement numbers for all samples.

### 2.7. Measurement of Cytokine Induction

The experimental setup was chosen as described in the section Measurement of Transmembrane Potential and Activation of Caspase-3, except for treatment with chemotherapeutic agents. To analyze cytokine induction, supernatants were collected and analyzed for IL-6, IL-8, TNF and IL-10 using LEGENDplex^TM^ assays (Biolegend, San Diego, CA, USA) according to manufacturer’s instructions.

Intracellular levels of IL-6 and IL-8 were flow cytometrically determined in Fixable Aqua^−^ CD33^+^ cells using the BD Cytofix/Cytoperm^TM^ Fixation/Permeabilization Kit with an anti-IL-6-PE and anti-IL-8-PE antibody or mIgG1-PE as isotype control using a BD FACSCanto^TM^ II (all BD Biosciences).

### 2.8. Statistics

Unless otherwise specified, data are presented as mean with standard deviation, median and box plots with min/max whiskers. For combined data, the number of patients examined is noted accordingly. Statistical analyses were performed using GraphPad Prism 9.1.2 (GraphPad Software, San Diego, CA, USA). To analyze normality distribution, Shapiro–Wilk normality test was applied. Comparison of individual groups was done by a 2-tailed paired Student’s *t*-test or a Wilcoxon matched-pairs signed rank test, unpaired data were compared using a 2-tailed unpaired Mann–Whitney test or a Kruskal–Wallis test followed by Dunn’s multiple comparisons test. Association of mRNA and SFI levels as well as proportion of RANK^+^ cells and WBC counts were analyzed using Spearman’s rank correlation coefficient. Survival of AML patients was analyzed by Kaplan–Meier method with log-rank test to estimate survival differences between the groups. Cut-off values for separation of individuals into RANK^high^ and RANK^low^ were determined using receiver operating characteristic (ROC) analysis in JMP^®^ Pro 14.2 (SAS, Heidelberg, Germany) and were defined through value of highest Youden’s index. *p*-values of < 0.05 were considered statistically significant.

## 3. Results

### 3.1. RANK Is Expressed by AML Cells

**Figure 1:**
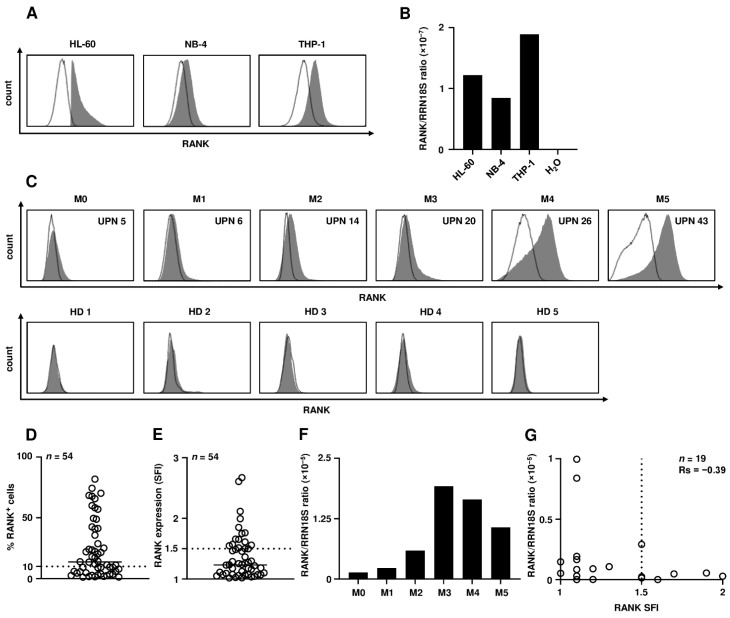
RANK expression in AML. (**A**) RANK surface expression on AML cell lines HL-60, NB-4 and THP-1 was analyzed by flow cytometry using the anti-RANK mAb FAB683P (shaded peaks) with mouse IgG1 serving as isotype control (open peaks). (**B**) *RANK* expression was assessed by quantitative PCR and abundance of *RANK* mRNA was calculated using delta-Ct method relative to *RRN18S* expression. (**C**) Exemplary results for RANK surface levels on PBMC of AML patients with different FAB types (top row) and on CD34^+^ hematopoietic progenitor cells contained in BM of healthy donors are shown (bottom row; shaded peaks, anti-RANK mAb FAB683P; open peaks, isotype control). Numbers in histograms represent uniform patient number (UPN) as shown in Table 1. (**D**,**E**) Combined results of RANK surface expression on primary AML cells showing (**D**) the proportion of RANK^+^ cells (solid line, median; dotted line, 10% RANK^+^ cells) and (**E**) SFI levels obtained by analysis of 54 patients (solid line, median; dotted line, SFI = 1.5 as defined threshold for surface positivity). Malignant cells in all patients with <85% blast count were defined based on blast selection markers of each individual patient. (**F**) *RANK* expression was assessed by quantitative PCR and abundance of *RANK* mRNA was calculated using delta-Ct method relative to *RRN18S* expression. Exemplary results for relative mRNA levels of PBMC from AML patients with different FAB types (M0, UPN 2; M1, UPN 11; M2, UPN 17; M3, UPN 22; M4, UPN 28; M5, UPN 49) are shown. (**G**) Relative *RANK* mRNA levels of different AML patients (*n* = 19) are plotted against RANK SFI values.

As a first step, we analyzed various AML cell lines to determine whether RANK is expressed on these malignant hematopoietic cells. Flow cytometric analyses of the AML cell lines HL-60, NB-4 and THP-1 revealed different levels of surface positivity (Figure 1A). *RANK* expression was also confirmed by determination of mRNA levels using quantitative PCR (Figure 1B). Next, we analyzed leukemic cells of AML patients and found that also primary AML cells express substantial levels of RANK on the cell surface (top row), whereas CD34^+^ hematopoietic progenitor cells contained in BM of healthy donors (bottom row) did not display relevant expression (Figure 1C). A high interindividual variation with regards to RANK expression was observed between the different patients; individual SFI levels as well as the proportion of RANK^+^ among leukemic cells and clinical characteristics of the patients are summarized in Table 1. Among the investigated patients, 31/54 (57%) showed RANK expression on at least 10% of the blasts (Figure 1D), and 19/54 (35%) expressed SFI levels of at least 1.5 as the defined threshold for surface positivity (Figure 1E). Expression was again confirmed by analysis of *RANK* mRNA levels using quantitative PCR (Figure 1F). No correlation between RANK mRNA and surface levels was observed in the patients, which points to posttranscriptional/posttranslational mechanisms that influence RANK surface expression (Figure 1G; Spearman correlation coefficient, Rs = −0.39). Notably, this phenomenon has also been observed for several other members of the TNF/TNFR superfamily including RANKL, GITR/L, OX40 and BAFF by us and other investigators [14,25,26,27]. Altogether, our data demonstrate that RANK is expressed in a substantial proportion of AML cases.

### 3.2. RANK Induces Cytokines Involved in Pathophysiology and Promotes Metabolic Activity of AML Cells

**Figure 2:**
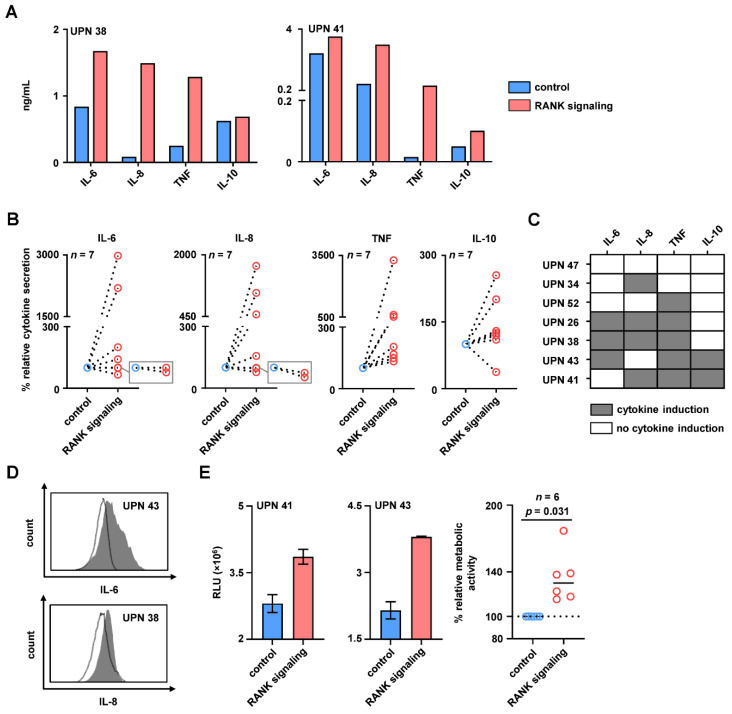
Induction of cytokines and metabolic activity upon RANK signaling in primary AML cells. PBMC of AML patients were cultured with Fc-hRANKL or rhIgG1-Fc as isotype control. (**A**–**C**) The levels of IL-6, IL-8, TNF and IL-10 in culture supernatants were determined by LEGENDplex^TM^ assay after 24 h. (**A**) Exemplary data on the induction of IL-6, IL-8, TNF and IL-10 in culture supernatants of 2 AML patients are depicted. (**B**) Combined data of the cytokine release in *n* = 7 patients normalized to the isotype control are shown and individual patients are connected by dotted lines. (**C**) Patient-specific cytokine induction pattern upon RANK signaling is shown as heatmap representation (grey, cytokine induction; white, no cytokine induction). (**D**) Intracellular cytokine levels of IL-6 and IL-8 in 2 exemplary AML patients were analyzed by flow cytometry (shaded peaks, intracellular IL-6/IL-8; open peaks, isotype control). (**E**) Metabolic activity of leukemic cells was determined by CTG assay after 24 h. Results of representative experiments (left 2 panels) as well as combined results (right panel) obtained in independent experiments with leukemic cells of *n* = 6 AML patients are depicted (solid line, median; dotted line, 100% relative metabolic activity). Numbers in panels, heatmap and histograms represent uniform patient number (UPN) as shown in Table 1.

To determine whether RANK transduces activating signals into AML cells, primary leukemic cells of patients were incubated with the RANK agonist Fc-hRANKL or isotype control for 24 h, followed by analysis of cytokine levels in the culture supernatants using LEGENDplex^TM^ assays. Stimulation of RANK was found to induce the release of IL-6, IL-8, TNF and IL-10 by AML cells (Figure 2A,B). These cytokines were previously described to contribute to the cytokine milieu associated with AML pathophysiology and thus create an environment conductive for AML cells. Substantial interindividual differences concerning the release of cytokines by AML cells upon RANK signaling were observed: For none of the 7 investigated samples, release of all four cytokines was observed, whereas at least 1.5-fold increased release of IL-6, IL-8, TNF and IL-10 compared to the control was observed in 3, 4, 5 and 2 patient samples, respectively (Figure 2C). RANK may thus (variably) contribute to an AML-associated cytokine milieu. Analysis of intracellular IL-6 and IL-8 levels by flow cytometry and gating for Fixable Aqua^−^/CD33^+^ cells ascertained that in fact the leukemic cells among patient-PBMC produced the respective cytokines upon signals transduced by RANK (Figure 2D). 

Next, we determined whether and how RANK signaling affected the viability of AML cells. We found that stimulation of RANK significantly increased metabolic activity as indicator of cell proliferation and viability in the primary AML cells (Figure 2E; *p* = 0.031). This indicates that RANK signaling transduces activating signals into primary AML cells that promote release of cytokines involved in pathophysiology as well as AML cell metabolic activity/viability.

### 3.3. RANK Mediates Chemotherapy Resistance of AML Cells

**Figure 3:**
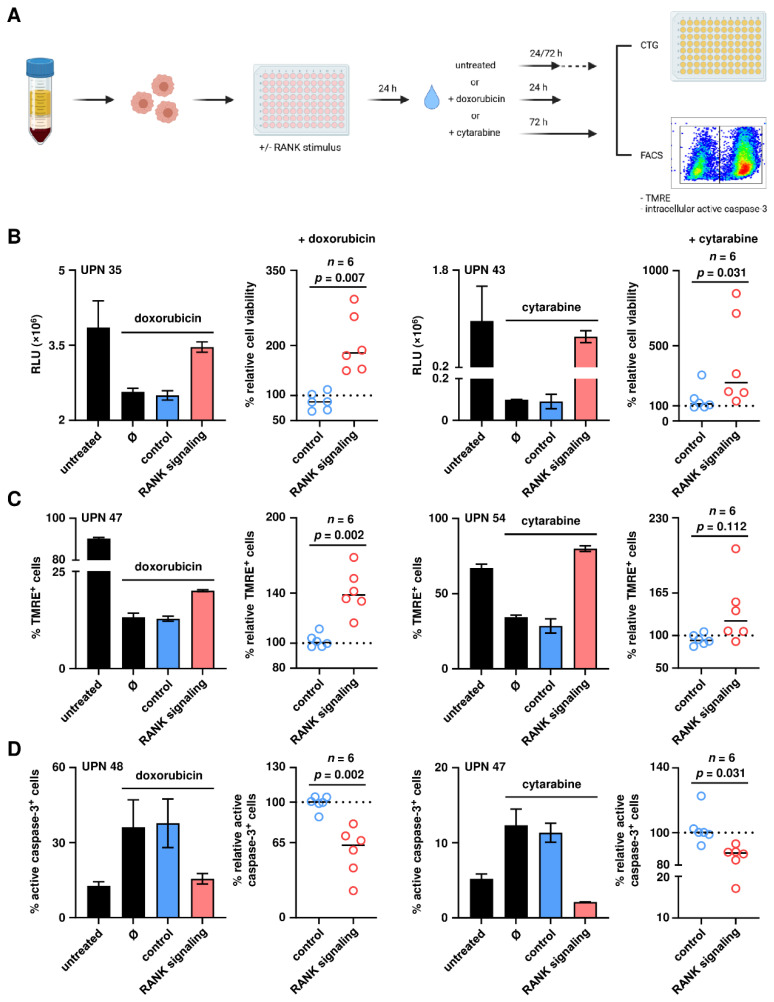
Resistance of primary AML cells to chemotherapeutic agents upon RANK signaling. (**A**) Schematic workflow for the analysis of resistance to doxorubicin and cytarabine of primary AML cells upon stimulation of RANK. (**B**) Cell viability was determined by CTG assay. Results of one representative experiment (bars) and combined results obtained in independent experiments with leukemic cells of *n* = 6 AML patients are shown (dot plots; solid lines, median; dotted lines, 100% relative cell viability; Ø, samples only treated with the respective chemotherapeutic agent). For combined results, data were normalized to samples treated with the respective chemotherapeutic agent (Supplementary Figure S2) and relative cell viability was calculated. (**C**) Mitochondrial membrane potential of AML cells was analyzed by staining with TMRE using flow cytometry. Results of one representative experiment (bars) and combined results obtained in independent experiments with leukemic cells of *n* = 6 AML patients are shown (dot plots; solid lines, median; dotted lines, 100% relative mitochondrial membrane potential; Ø, samples only treated with the respective chemotherapeutic agent). For combined results, data were normalized to samples treated with the respective chemotherapeutic agent (Supplementary Figure S2) and relative mitochondrial membrane potential was calculated. (**D**) Active caspase-3 in viable AML cells was determined intracellularly by flow cytometry. Results of one representative experiment (bars) and combined results obtained in independent experiments with leukemic cells of *n* = 6 AML patients are shown (dot plots; solid lines, median; dotted lines, 100% relative active caspase-3^+^ primary AML cells; Ø, samples only treated with the respective chemotherapeutic agent). For combined results, data were normalized to samples treated with the respective chemotherapeutic agent (Supplementary Figure S2) and relative percentage of active caspase-3^+^ primary AML cells was calculated. Numbers in panels represent uniform patient number (UPN) as shown in Table 1.

Based on our finding that RANK affects AML cell viability, we next analyzed whether signaling via RANK also affects resistance of the leukemic cells to treatment with doxorubicin and cytarabine, the most commonly used chemotherapeutics utilized for treatment of AML. A schematic representation of the experimental approach is given in Figure 3A. PBMC of AML patients were incubated with Fc-hRANKL or isotype control for 24 h. Then cells were treated with either doxorubicin and cytarabine for additional 24 h and 72 h, respectively. Thereafter, the consequences of RANK signaling for treatment resistance in terms of ATP content, mitochondrial membrane potential and apoptosis of the leukemic cells were analyzed by CTG assays, flow cytometry for TMRE staining and analysis of intracellular caspase-3 activity, respectively. Analyses of ATP levels revealed that RANK signaling significantly protected the AML cells from drug-induced cell death induced by either chemotherapeutic agent (Figure 3B; doxorubicin, *p* = 0.007; cytarabine, *p* = 0.031). In addition, signals transduced by RANK prevented the effects of treatment on AML cell mitochondrial membrane potential: upon treatment with doxorubicin, RANK signaling significantly increased the proportion of TMRE^+^ cells (*p* = 0.002). Similar effects were observed upon exposure of the AML cells to cytarabine, which however closely failed to reach statistical significance (Figure 3C; *p* = 0.112). Analyses of downstream apoptotic events, i.e., treatment-induced activation of caspase-3, an effector caspase with a central role in the process of cell apoptosis, revealed that RANK signaling also reduced the cleavage of procaspase-3 into its active form upon chemotherapeutic treatment: a significant reduction of active caspase-3 levels upon RANK stimulation was observed in the presence of both doxorubicin and cytarabine (Figure 3D; *p* = 0.002 and 0.031, respectively). Similar but less pronounced results were observed in all assay systems when non-clustered trimeric soluble hRANKL (sRANKL) instead of multimeric Fc-hRANKL was used to induce signals by RANK (Supplementary Figure S1), in line with data, that for TNF family members in general and RANK in particular multimerization of ligands is required to obtain optimal signaling efficacy [28,29]. Altogether, our findings demonstrate that in AML cells signaling via RANK confers resistance to chemotherapy.

### 3.4. RANK Expression Is Associated with Dismal Survival of AML Patients 

**Figure 4:**
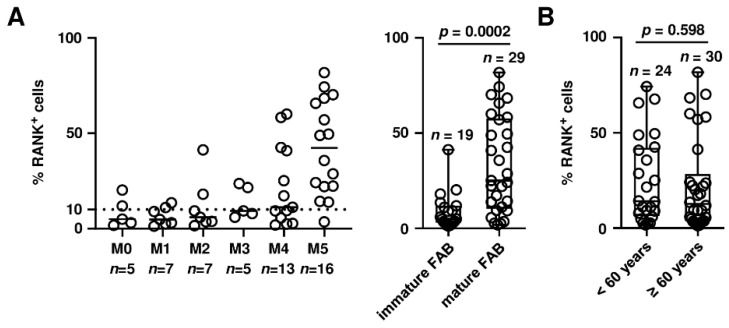

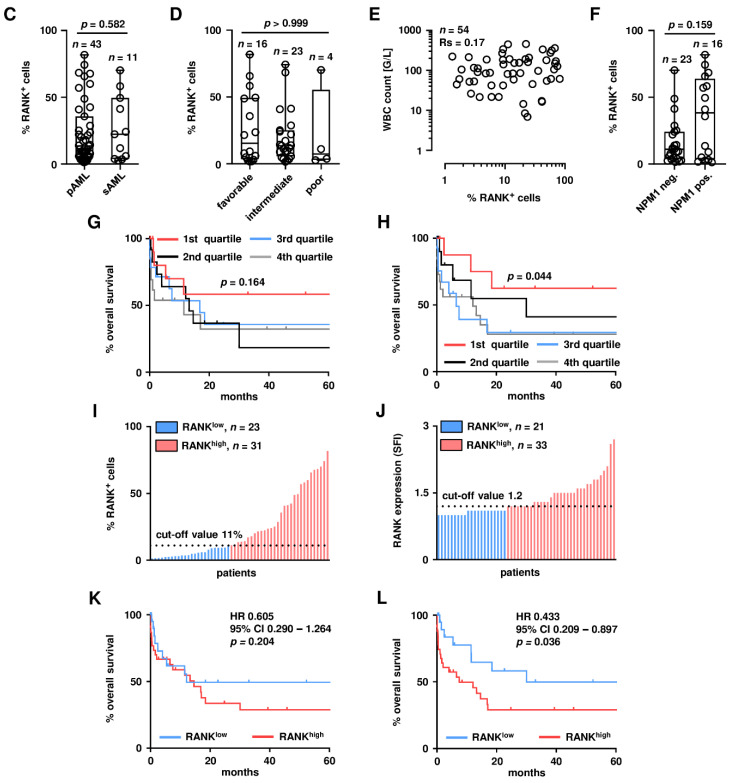
Association of RANK expression with clinical parameters and prognostic evaluation of RANK in AML. (**A**) Proportion of RANK^+^ cells in primary AML samples are grouped according to the individual FAB type (left panel) and in immature (M0-2, *n* = 19) versus mature (M4-5, *n* = 29) FAB types (right panel; solid lines, median; dotted line, 10% RANK^+^ cells). (**B**-**D**) Distribution of RANK expression (percentage of positive cells) in (**B**) AML patients below and above 60 years (<60 years, *n* = 24; ≥60 years, *n* = 30), (**C**) primary (pAML, *n* = 43) and secondary (sAML, *n* = 11) cases and (**D**) NCCN risk groups (favorable, *n* = 16; intermediate, *n* = 23; poor, *n* = 4) is shown. (**E**) Proportion of RANK^+^ cells in 54 primary AML samples are plotted against WBC counts. (**F**) AML cases positive (*n* = 16) or negative (*n* = 23) for *NPM1* mutations are shown. (**G**,**H**) Overall survival of 54 AML patients grouped into quartiles according to (**G**) percent RANK^+^ cells and (**H**) SFI levels are depicted (first to fourth quartile, low to high proportion of RANK^+^ cells and SFI levels, respectively). (**I**,**J**) Separation into RANK^low^ and RANK^high^ patient groups using the cut-off value of (**I**) 11% positive cells and (**J**) an SFI of 1.2 is shown. (**K**,**L**) Overall survival of patients assigned to the RANK^low^ and RANK^high^ groups based on the cut-off value of (**K**) 11% positive cells and (**L**) an SFI of 1.2 (HR, hazard ratio; CI, confidence interval).

Based on our findings on the role of RANK in chemotherapy resistance, we next analyzed whether RANK expression on AML cells correlates with clinical characteristics and survival of patients. As a first step, we correlated RANK positivity with morphological characteristics according to the French-American-British (FAB) classification. The fractions of samples expressing at least 10% RANK on their surface among patients with different FAB types were as follows: M0, 2 of 5 (40%); M1, 2 of 7 (29%); M2, 2 of 7 (29%); M3, 2 of 5 (40%); M4, 7 of 13 (54%); M5, 15 of 16 (94%). A significant association of RANK expression with leukemia cell maturity, i.e., specimen from AML patients with differentiated FAB types (M4-M5) compared to M0-M2 subclasses was observed (Figure 4A; *p* = 0.0002).

No clear correlation of RANK expression with age (Figure 4B; *p* = 0.598) and primary versus secondary AML (Figure 4C; *p* = 0.582) was observed. Likewise, no correlation of RANK expression with the categorization of patients based on their genetic profile according to the National Comprehensive Cancer Network (NCCN) risk score into three different risk-groups (Figure 4D; *p* > 0.999) [30], white blood count (Figure 4E; Rs = 0.17) and recurrent genetic alterations such as nucleophosmin 1 (*NPM1*) mutations (Figure 4F; *p* = 0.159) was observed. To study whether RANK expression correlates with survival of AML patients, we grouped patients into quartiles according to the proportion of RANK^+^ cells (Figure 4G) or SFI levels (Figure 4H) and assessed overall survival (OS) in each group using Kaplan-Meier analysis. A tendency towards longer OS was observed when patients were grouped according to the percent RANK^+^ cells (Figure 4G; *p* = 0.164), while OS was significantly longer when SFI levels were used for determination of RANK positivity (Figure 4H; *p* = 0.044). Due to the substantial interindividual variation of RANK expression among AML patients, for subsequent analysis, predicted cut-off values were estimated by ROC analysis. A cut-off value of 11% (Figure 4I) and an SFI of 1.2 (Figure 4J) separated all AML patients in low and high expressing cases. OS tended to be longer in RANK^low^ patients than in high expressing cases when a cut-off value of 11% RANK expression was used (Figure 4K; hazard ratio 0.605, *p* = 0.204). A clear and statistically significantly longer OS was observed when comparing the groups below versus above an SFI of 1.2 (Figure 4L; hazard ratio 0.433, *p* = 0.036). These results are in line with the functional role of RANK observed in our antecedent experiments and identify a prognostic relevance of RANK in AML. 

## 4. Discussion

The TNF superfamily member RANK and its cognate ligand are key players in bone metabolism [12,13]. RANK is expressed on osteoclast precursors, while its ligand RANKL is expressed in membrane-bound form on and cleaved from the surface of osteoblasts [31,32,33]. Receptor-ligand interaction causes the differentiation of progenitor cells into active osteoclasts, which then degrade bone. Beside its role in bone metabolism, available data document a role of RANK also in various other cellular functions and the pathophysiology of diseases such as thermoregulation and metastatic spread of different cancer entities mediation [14,15,16,17,18,19,20,21,22,34].

Here we report that RANK is functionally expressed on leukemic cells in a high number of AML cases, with expression being associated with a mature phenotype. Our ex vivo study using leukemia cells of AML patients identified RANK as potent influencer of cellular function and thus potential target for therapeutic approaches: upon RANK stimulation we observed induction of the cytokines IL-6, IL-8, TNF and IL-10, which can act as autocrine/paracrine growth and survival factors in AML and contribute to disease pathophysiology [35,36,37,38]. Among others, Hou et al. [39] demonstrated that knockout of IL-6 in bone marrow stromal cells increased chemosensibility of AML cells, and likewise IL-8 was reported to promote proliferation and chemotherapy resistance [40]. IL-10 was shown to positively affect cell survival via autocrine mechanisms and upregulation of E-Cadherin acting as survival factor [41]. TNF was, among others, suggested to form a positive feedback loop with NF-κB resulting in enhanced leukemia progression [42], and high TNF serum levels correlate with poor event-free and overall survival of AML patients [35]. In line, analyses of cell viability, mitochondrial membrane potential and activation of caspase-3 using primary leukemic cells revealed that RANK signaling increases cell viability and protects primary cells from apoptosis. Notably, this also held true upon exposure to doxorubicin or cytarabine, the most commonly used cytostatic compounds in AML treatment. 

Our ex vivo analyses, enabled by the use of AML patient material, are particularly well suited when it comes to translation of results into clinical application in humans when compared to studies in murine models, as TNFR family members reportedly can mediate different effects in mice and men [43,44,45]. To ensure that our results were indeed due to effects in leukemic cells, only patient samples with ≥85% blast count were used in functional analyses. This served to avoid artifacts arising from purification procedures. In addition, confirmation that effects occurred specifically in leukemia cells was provided by intracellular flow cytometry analyses where leukemic cells were selected by staining with appropriate surface markers. 

The functional relevance of RANK in AML identified in our ex vivo analyses was further corroborated by our finding that RANK surface expression correlated with dismal disease outcome: a strong correlation of RANK positivity with shorter OS was detected, which also identified RANK surface expression as potential novel prognostic marker in AML. Notably, the association with disease course was clearly significant despite the relatively small cohort of 54 patients included in our study. This is of particular interest considering that no correlation of gene expression of RANK in AML cells with overall survival of patients was observed upon analysis of the “Acute Myeloid Leukemia (OHSU, Nature 2018) Whole-exome sequencing of acute myeloid leukemia samples from the Beat AML program” dataset from cBioPortal for Cancer Genomics. However, it should be considered that the public database only allows for correlative analyses of gene expression levels, but not for protein levels as in our study. Taking into account our finding that RANK mRNA and protein levels in AML cells do not correlate, not only the seeming discrepancy is explained, but also the relevance of analysis of protein expression as conducted in our study is underlined. Nevertheless, future analyses in larger cohorts are warranted to confirm our findings and to validate the utility of RANK as predictor of prognosis in AML.

RANK is known to interact with various TNFR-associated factor (TRAF) molecules (including TRAF 6) that are linked, among others, to activation of the transcription factor NF-κB [46]. The latter is generally considered to exert pro-survival effects, for example by inducing expression of caspase inhibitors (inhibitors of apoptosis, IAPs) and mitochondria-mediated cell death inhibitors such as B-cell lymphoma-extra large (Bcl-xL) [47,48], and reportedly can promote oncogenesis in mammalian systems [49]. Prevention of cell death through induced inhibitors upon activation of NF-κB might also underlie the protective effects of RANK signaling observed with the AML patient samples in our study. In line, recent findings on resistance of AML cells e.g., upon exposure to a multikinase inhibitor targeting fms such as tyrosine kinase 3 (FLT3) as well as IL-1 receptor-associated kinase 1 and 4 (IRAK1/4) point to a mechanism associated with NF-κB [50]. Together with our results, these findings further highlight the potential of therapeutic RANK modulation to sensitize malignant cells to treatment. This could be achieved by inhibiting of RANK-RANKL interaction and thus RANK signaling into AML cells, e.g., using denosumab, a clinically approved neutralizing RANKL antibody that was found to improve disease-free survival in breast cancer patients [51]. This is even more since AML arises in the BM where (i) various cells abundantly express RANKL (e.g., [12,14,16]) and since (ii) the tightly controlled microenvironment plays an important role in transformation and development of malignant hematopoietic disease as well as chemotherapy resistance [52,53]. 

## 5. Conclusions

In conclusion, we provide the first evidence that RANK is expressed in AML and mediates resistance to chemotherapy. Moreover, the association of RANK expression with dismal disease course identifies RANK as potential “functional” prognostic marker and putative target for therapeutic intervention to improve treatment response of patients.

## Figures and Tables

**Table 1:** Patient characteristics and RANK expression.

UPN	RANK		FAB	Age	Sex	PBB	Karyotype	WBC	Hb	Plt
	[%]	[SFI]		[Years]		[%]		[G/L]	[G/dL]	[G/L]
1	1.7	1.0	M0	46	M	97	46,XY	60.6	7.0	39
2	11.9	1.5	M0	83	M	90	48,XY,+X,+13	191.6	8.9	59
3	3.1	1.8	M0	68	M	93	ND	52.8	10.5	114
4	4.9	1.6	M0	65	M	85	46,XY	186.3	9	11
5	20.2	1.3	M0	90	F	97	complex	14.5	8.1	586
6	11.0	1.2	M1	40	M	100	complex	81.3	10.8	51
7	13.5	1.7	M1	69	M	86	46,XY	84.1	6.7	322
8	4.8	1.0	M1	21	F	95	46,XX; 46,XX,del(9)(q13q22)	84.0	7.1	30
9	2.6	1.1	M1	56	F	56	46,XX	52.0	12.1	9
10	3.0	1.1	M1	77	M	87	46,XY	116.0	7.3	57
11	9.0	1.5	M1	50	F	93	46,XX	267.8	8.0	18
12	1.3	1.1	M1	64	F	98	ND	222.2	9.2	44
13	1.6	1.1	M2	88	M	29	ND	45.4	8.9	81
14	18.0	1.1	M2	68	M	96	46,XY	85.5	9.5	146
15	9.3	1.2	M2	64	M	82	complex	338.5	8.1	19
16	5.9	1.1	M2	60	M	95	46,XY,+14	42.0	10.1	57
17	41.3	1.1	M2	71	F	89	47,XX,+11	16.4	8.6	18
18	3.8	2.6	M2	79	F	69	46,XX	21.5	7.0	14
19	3.5	1.1	M2	67	F	94	complex	112.7	10.6	137
20	21.5	1.3	M3	46	M	87	46,XY,t(15;17)(q22;q11~21)	8.42	9.9	40
21	23.6	1.5	M3	65	M	70	46,XY,t(15;17)(q22;q12)	7.0	9.7	27
22	6.0	1.1	M3	29	M	93	46,XY,t(15;17)(q22;q12)	21.6	7.1	61
23	9.3	1.0	M3	58	F	96	46,XX,t(15;17)(q22;q12)	42.1	8.4	17
24	7.9	1.0	M3	46	F	42	46,XX,t(15;17)(q24.1;q21.2)	21.6	7.1	14
25	2.5	1.0	M4	30	F	90	complex	214.0	6.4	10
26	58.3	1.7	M4	64	F	91	46,XX	61.5	7.2	100
27	5.6	1.1	M4	71	M	97	47,XY,+11	87.1	7.5	23
28	9.4	1.0	M4	76	F	94	complex	140.9	12.0	70
29	9.3	1.2	M4	85	M	92	ND	183.2	8.9	64
30	11.2	1.1	M4	45	F	97	46,XX,t(1;3)(p36;q21)(22)	448.3	6.6	36
31	1.9	1.0	M4	62	M	91	complex	104.7	6.5	34
32	17.2	2.1	M4	83	F	95	46,XX,add(14)(p11); 46,XX	155.8	11.6	144
33	25.1	1.2	M4	36	M	95	46,XY	207.4	6.1	55
34	60.0	1.9	M4	67	F	86	ND	315.9	8.2	34
35	42.5	1.6	M4	57	M	87	ND	333.7	9.4	293
36	40.9	1.3	M4	54	F	91	46,XX	17.2	10.6	167
37	2.7	1.0	M4	57	M	14	45,XY,inv(3)(q21.3q26.2),−7	26.2	10.4	252
38	57.0	1.6	M5	69	F	95	ND	274.9	7.1	47
39	3.5	1.1	M5	72	M	97	47,XY,+8; 46,XY	90.3	8.9	79
40	22.3	1.5	M5	65	M	91	ND	151.0	7.9	151
41	70.2	1.3	M5	76	M	93	complex	169.3	9.9	26
42	35.7	1.2	M5	54	M	89	46,XY,del(9)(q13q22)	97.1	8.1	73
43	74.3	2.0	M5	37	F	85	ND	126.8	9.7	41
44	81.8	1.2	M5	81	M	93	46,XY	61.3	11.7	72
45	14.3	1.2	M5	23	M	92	48,XY,+8,+13; 46,XY	153.5	6.7	44
46	28.7	1.2	M5	35	F	83	46,XX	45.4	8.9	81
47	65.7	1.5	M5	53	M	85	46,XY	105.6	8.1	35
48	49.5	1.5	M5	48	M	95	46,XY	54.6	6.8	190
49	22.0	1.3	M5	70	M	90	46,XY	190.9	7.1	65
50	48.9	1.4	M5	32	M	98	complex	179.3	3.3	80
51	24.1	1.5	M5	71	M	94	complex	161.1	8.6	61
52	68.4	1.8	M5	68	M	95	46,XY	148.7	9.1	134
53	13.9	1.0	M5	41	F	92	46,XX	59.9	8.9	34
54	67.7	2.7	ND	49	M	96	ND	316.0	7.1	80

UPN, uniform patient number; SFI, specific fluorescence index; FAB; French-American-British classification; F, female; M, male; PBB, peripheral blood blasts among nucleated cells; WBC, white blood count; Hb, hemoglobin; Plt, platelets; ND, not determined. Complex karyotypes were defined as having 3 or more chromosome aberrations.

## Data Availability

The data presented in this study are available on request from the corresponding author. The data are not publicly available due to the used patient material.

## Supplementary Materials

The following are available online at https://www.mdpi.com/article/10.3390/cancers13236122/s1, Figure S1: Effects of RANK signaling in primary AML cells treated with chemotherapeutic agents using sRANKL, Figure S2: Extended data on resistance to doxorubicin and cytarabine upon RANK signaling in primary AML cells.
