# Supplementary material for: Receptor Activator of NF-κB (RANK) Confers Resistance to Chemotherapy in AML and Associates with Dismal Disease Course
Source: Cancers (Basel). 2021 Dec 4;13(23):6122. doi: 10.3390/cancers13236122 (PMC8657109; doi:10.3390/cancers13236122)
Supplement: Supplementary file 1 [file cancers-13-06122-s001.zip › cancers-1474198-supplementary.pdf]

cancers

MDPI

Supplementary Materials

Receptor Activator of NF-κB (RANK) confers resistance to chemotherapy in AML and associates with dismal disease course

Kim L. Clar, Lisa M. Weber, Bastian J. Schmied, Jonas S. Heitmann, Maddalena Marconato, Claudia Tandler, Pascal Schneider and Helmut R. Salih

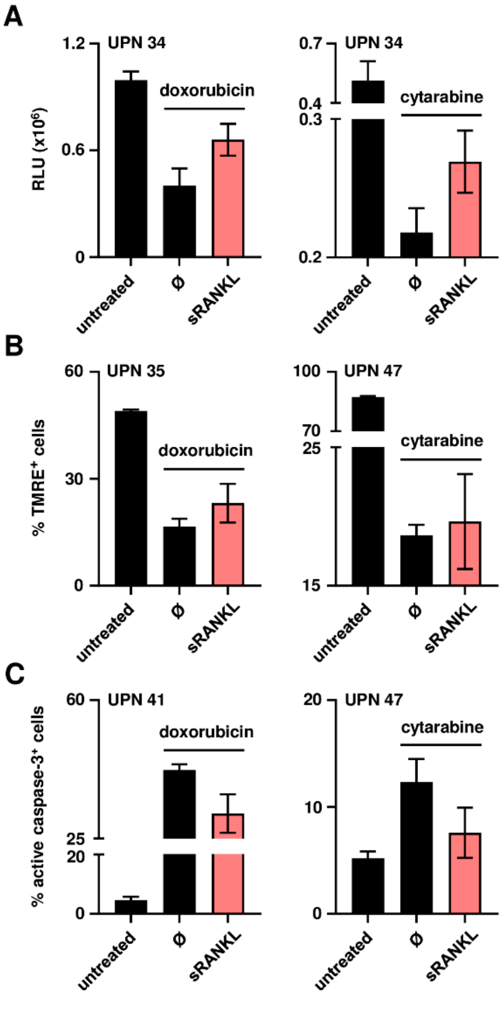

Figure S1. Effects of RANK signaling in primary AML cells treated with chemotherapeutic agents using sRANKL. (**A**) ATP levels, (**B**) mitochondrial membrane potential and (**C**) cell death were analyzed by CTG assays, flow cytometry for TMRE staining and analysis of intracellular caspase-3 activity, respectively, in primary AML cells upon RANK signaling using non-clustered trimeric sRANKL (0.5 µg/mL) and treatment with either chemotherapeutic agent (Ø, samples only treated with the respective chemotherapeutic agent). Numbers in panels represent uniform patient number (UPN) as shown in Table 1.

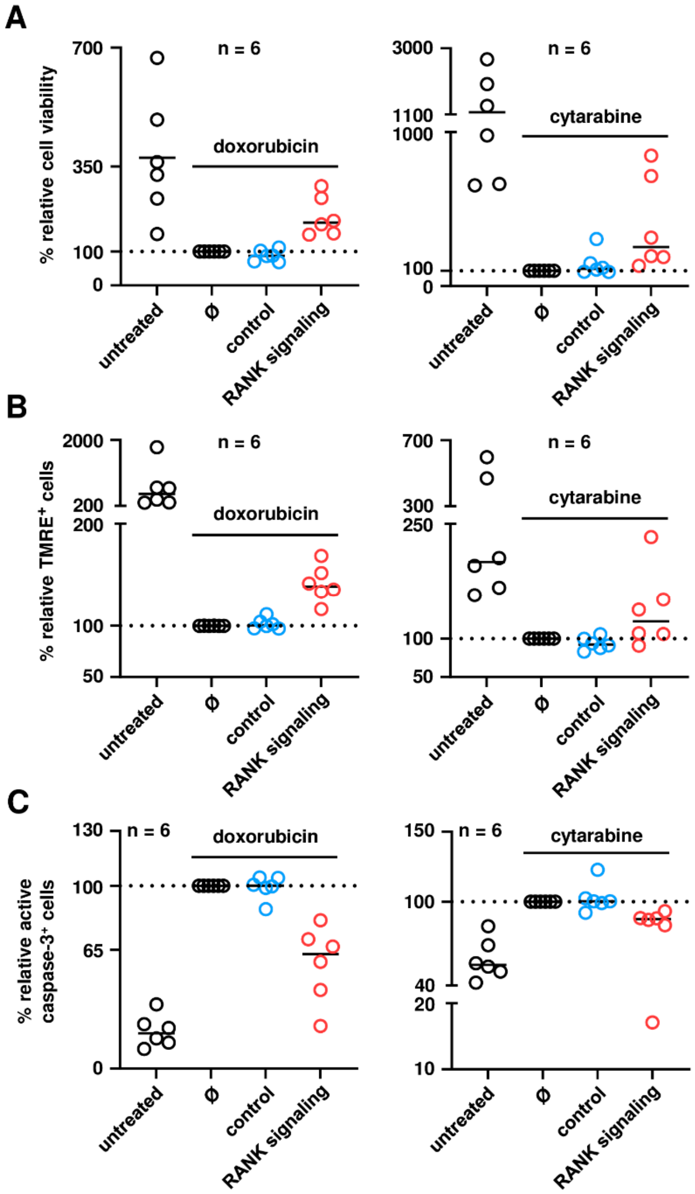

Figure S2. Extended data on resistance to doxorubicin and cytarabine upon RANK signaling in primary AML cells. Additional data on untreated primary AML cells and samples solely treated with the respective chemotherapeutic agent (Ø) are shown. Combined results obtained in independent experiments with leukemic cells of n = 6 AML patients upon RANK signaling and treatment with either chemotherapeutic agent by (**A**) CTG assay, (**B**) flow cytometry for TMRE staining and (**C**) analysis of intracellular caspase-3 activity are depicted (solid lines, median; dotted lines, 100% relative cell viability, mitochondrial membrane potential and active caspase-3^{+} primary AML cells, respectively). Data were normalized to samples treated with the respective chemotherapeutic agent.
